# Relationship between Patient Experience Scores and Health Insurance

**DOI:** 10.3390/healthcare10112128

**Published:** 2022-10-26

**Authors:** Walter Markowitz, Khadeja Kausar, Edward Coffield

**Affiliations:** 1 Department of Population Health, Hofstra University, Hempstead, NY 11549, USA; 2 Maimonides Medical Center, Brooklyn, NY 11219, USA

**Keywords:** patient experience, health insurance, value-based

## Abstract

(1) Background: the patient experience may be a performance indicator in value-based reimbursement. Accordingly, providers have an incentive to understand factors that affect their patients’ experiences. This study evaluated the relationship between health insurance type and patient experience ratings. (2) Methods: individual-level demographic, health/healthcare, and patient experience data were extracted from the Full-Year Consolidated Data File of the 2019 Medical Expenditure Panel Surveys. A logistic regression was used to evaluate whether how persons—included in this study’s analytic sample (aged 18 and over with complete covariate information)—rated the healthcare they received from all their providers was associated with their health insurance types controlling for covariates. (3) Results: relative to people 18–64 years of age with private health insurance, people 18–64 years of age without health insurance were less likely to rank their healthcare as a 9 or 10—where a 10 indicates the best possible care—(OR: 0.69; *p* = 0.015) while people aged 65 years or over with Medicare (OR: 1.34; *p* = 0.002) or with Medicare/private health insurance (OR: 1.48; *p* < 0.001) were more likely to rank their healthcare as a 9 or 10. (4) Conclusions: Select health insurance types were associated with how patients rate their healthcare. Stakeholders could use this information to create programs aimed to improve patient experience.

## 1. Introduction

Payment mechanisms may be used to incentivize providers and hospitals to focus on healthcare improvements [1]. Such mechanisms are included in many value-based payment models [2,3,4], models that are increasingly used in healthcare [4,5,6] and will likely continue. For instance, the Centers for Medicare and Medicaid Services Innovation Center has a goal of having all Medicare Part A and Part B beneficiaries and most Medicaid beneficiaries in “care relationships” that include accountability for cost and quality [7]. One area on which value-based payment models may focus is patient experience [8,9,10]. The inclusion of patient experience metrics in value-based payment models should encourage providers to expand their quality improvement process beyond the traditional clinical-based metrics—or a technical intervention approach that includes such elements as actual treatments, surgeries, medications administered, instructions, information, and other metrics—to one that also incorporates processes to measure the subjective nature of patient-based metrics.

Press (2006) stated that many healthcare professionals, both clinical and non-clinical, view patient satisfaction/the perceived patient experience as “…a happy camper index...” [11] (p. 2). Press (2006) describes what was called the “service” approach (or ‘smile school’), which describes elements contained in the guest relations programs emanating from the 1980s. Since patient experience metrics are based upon patients’ subjective feelings, they have been often viewed as having inconsequential effects on the quality of care or clinical outcomes, differing from the traditional, clinical-based performance metrics. However, Press (2006) supports the premise that patients’ perception of care indeed plays a crucial role in understanding what should be considered in a model for quality of care [11]. Patient outcomes are improved when patients trust their physician, when they believe that the physician listens to them and when the physician can effectively communicate with them in a manner exhibiting competence, empathy, and compassion [11]. This trust results in improved patient compliance with treatment plans [11]. The sharing of information between patient and physician during an effective consent process has the desired result of a mutually agreed upon plan, often considered essential to good care [12]. 

The inclusion of patient satisfaction/experience data to more broadly define quality and include them for purposes of reimbursement has had its opponents and concerns. Conflicting outcomes, utilization, and cost data have been evident since the later launching of value-based reimbursement models. In an early study, Fenton et al. (2012) reported in their conclusions: “…higher patient satisfaction was associated with less emergency, department use but with greater inpatient use, higher overall health care and prescription drug expenditures, and increased mortality” [13] (p. 405). In a review of 55 studies, Doyle, Lenox and Bell (2012) analyzed possible relationships between patient experience with safety and effectiveness [14].

Patient experience has become a key metric in assessing provider performance. For instance, Medicare’s hospital value-based reimbursement model includes the patient experience-based Person and Community Engagement domain as 25% of hospitals’ Total Performance Scores [10,15]. A number of studies have examined possible factors that may influence patient experience metrics, including factors such as race [16,17,18,19,20], geography [16,18], ethnicity [16,17,19,21], and language [19,22]. Patients’ experiences may also differ based upon their insurance type [21,23].

Using a nationally representative sample, we extend the literature on how patients rate their care experiences by evaluating whether a summary measure of patient experience (how patients rate the healthcare from all their providers) differs based upon patients’ insurance types. Health insurance type may be associated with patient experience scores through multiple avenues. For instance, managed-care elements such as provider networks and utilization management found in Medicare Advantage, Medicaid managed care plans, and private-based managed health insurance plans may cause patients to evaluate their care differently than patients not subject to such managed care elements (e.g., people with traditional government-based Medicare). Similarly, different reimbursement mechanisms (e.g., whether care management is provided) could influence the clinical- and administrative-based experiences patients have with their providers. The purpose of this study was to evaluate whether an association was present between health insurance type and patient experience measured by how patients rate their healthcare from all their providers. A next research step would be to explore the possible underlying mechanisms that may help explain any found associations.

There is a need to evaluate whether a relationship exists between patient experience scores and the insurance type or payer. First, payers help determine the structure of value-based payment systems. Knowing whether patient experience ratings differ by insurance type allows providers to have vital information in reimbursement negotiations with payers on value-based payment models. Second, examining the relationship between specific patient experience metrics and patient insurance type will highlight any possible patient experience areas where providers could consider implementing performance improvement interventions.

## 2. Materials and Methods

Individual-level data were extracted from the 2019 Full-Year Consolidated Data File of the Medical Expenditure Panel Survey (MEPS) [24,25]. MEPS is an annual nationwide survey administrated by the Agency for Healthcare Quality and Research, United States Department of Health and Human Services. This study only used data from the 2019 MEPS survey. Respondents of the 2019 MEPS—including individuals, families, medical providers, and employers—completed several surveys while in the MEPS sample. MEPS surveys collect information on the use, frequency, and cost of healthcare services as well as who pays for these services and other individual-level health and demographic factors. The MEPS survey includes a module that evaluates the experiences of patients within healthcare. This module contains questions from the Consumer Assessment of Healthcare Providers and Systems (CAHPS) health plan survey, one of a series of quality of care surveys sponsored by the Agency for Healthcare Research and Quality (AHRQ) to measure quality from a consumer perspective [25] (p. C-52).

All data used in the analyses were extracted from MEPS. The primary variables of interest were survey respondents’ answers to the health insurance question and then the CAHPS question, which asked respondents to rate their healthcare from their doctors/providers. Among respondents to whom the healthcare rating question was applicable, they were asked to rate the healthcare “from all doctors and other health care providers” on a scale of 0–10, where 10 indicated the “best care possible” and 0 indicated the “worst care possible” [25] (p. C-53). For this study, top box scoring was used for the healthcare rating question; this entailed assigning a value of 1 to the top score (9 or 10) and a value of 0 to the other possible responses. The top box scoring method is highlighted in CAHPS material [26] and in others [16,22,27,28]; the score from this method illustrates the percentage of respondents who selected the top outcome(s) for the survey question. In this study, as illustrated in an instruction manual for CAHPS [26] for global score measures and as recommended by the American Institute for Research (AIR) in a report for the Robert Wood Johnson Foundation (RWJ) for doctor rating questions, the top box was defined as a score of 9 or 10 [27]. The AIR/RWJ recommendation is based upon the combination providing comparability across respondents who vary by socioeconomics and sociodemographic factors [27].

Respondents’ health insurance types were based upon a yearly summary variable (INSURCyy) within MEPS that classified insurance into a series of mutually exclusive health insurance types as selected by MEPS [25]. In these mutually exclusive groups, preference is provided to private health insurance and TRICARE, which is classified as private health insurance in this MEPS measurement variable [25]. The health insurance types were: for persons under the age of 65: private, public only, or uninsured. For persons 65 years of age and older, the health insurance types were: Medicare only, Medicare and private, Medicare and other public, uninsured, and no Medicaid, but other public [25]. In addition to the CAHPS and health insurance type variables, covariates extracted from MEPS included: age (continuous, top-coded), gender, race/ethnicity, marital status, education level, poverty status, self-reported health status, and visits to a clinic/doctor’s office for care. While the health insurance type is partially determined by age in the United States entailing that age may be an effect modifier, the results were not stratified by age to maintain comparisons across all health insurance types. Variables may have been collected during different MEPS survey periods. In addition, some variables are based upon yearly information.

### Analytic Approach

The analytic sample consisted of MEPS respondents who were aged 18 years and older, completed the CAHPS module, and had complete variable data. First, summary statistics (means/percentages) were estimated for the variables for the overall sample and by health insurance group. Then, a multiple logistic regression model was used to evaluate whether how respondents rated their healthcare from all their providers was associated with their health insurance types. The dependent variable in this model was binary and indicated whether respondents rated their care as a 9 or 10 (top box score). The independent variables in the multiple logistic regression consisted of the health insurance types as well as the covariates. Survey commands within Stata 17 [29] were used to adjust for the complex survey design of MEPS. Results are considered significant at a 5% *p*-value.

## 3. Results

A primary factor that helped determine the MEPS respondents included in this study’s analytic sample was whether the MEPS respondents (unweighted sample (uws) = 28,512) had information for the healthcare rating variable; among all MEPS respondents, 55.51% (uws = 15,827) had a “not applicable” code on this variable while 1.22% (uws = 348) of MEPS respondents had a “cannot be computed” code for this variable. After adjusting for missing information (uws = 187), this study’s analytic sample consisted of those who rated the healthcare from their providers and had complete information for the other model variables (uws = 12,150). The majority of the respondents included in the analytic sample identified as female (57.20%), non-Hispanic White (70.26%), married (55.36%), and having completed at least some college (64.89%). The average years of age for respondents included in the analytic sample was 51.84. From a health insurance perspective, among the analytic sample respondents, 56.94% were 18–64 years of age and had private health insurance; 12.12% were 18–64 years of age and had public health insurance; 10.29% were 65 years of age or older and had Medicare; and 13.90% were 65 years of age or older and had Medicare/private health insurance. Additional insurance groups are listed in Table 1. While a large percentage of the analytic sample respondents perceived their health status as excellent (19.24%) and very good (35.39%), many also described their health as good (30.52%). The remaining respondents included in the analytic sample perceived their health as fair (11.76%) or poor (3.10%). Additional means/percentages are located in Table 1 as well as means/percentages by health insurance group. For instance, 60.18% of the respondents that were 65 years of age or older with Medicare rated the healthcare from their providers as a 9 or 10, whereas 62.36% of respondents 65 years of age and older with Medicare/private health insurance and 46.37% of respondents aged 18–64 years of age with private health insurance rated their healthcare from their providers as a 9 or 10.

Table 2 illustrates the results from the multiple logistic regression analysis. Select health insurance types were associated with how respondents included in the analytic sample rated healthcare from their doctors/providers. For instance, relative to the respondents in the analytic sample who were 18–64 years of age and had private health insurance, those who were 18–64 years of age and without health insurance were less likely (OR = 0.69, *p* = 0.015) to rate their healthcare from their providers a 9 or 10 on the 10-point scale. Conversely, respondents in the analytic sample who were 65 years of age or older and had Medicare only or Medicare/private health insurance were 1.34 (*p* = 0.002) and 1.48 (*p* < 0.001) times more likely, respectively, to rate their healthcare from their providers as a 9 or 10—on a 10 point scale where 10 indicates the best care possible—relative to those who were 18–64 years of age and had private health insurance.

Perceived health status was also associated with how respondents included in the analytic sample rated their healthcare from their doctors/providers. Respondents in the analytic sample who perceived their health to be excellent (OR = 3.98, *p* < 0.001), very good (OR = 2.34, *p* < 0.001), and good (OR = 1.48, *p* = 0.001) were estimated to be more likely to rate their healthcare from their providers with a top box score (a 9 or 10 on a 10-point scale where 10 indicates best possible care) relative to those who perceived their health to be poor. Age, gender identification, race/ethnic identification, and clinic and doctor’s office visits for care were also associated with how respondents rated their healthcare from their providers. For instance, respondents in the analytic sample who identified as non-Hispanic African American/Black were more likely to rate their healthcare from their providers with a top box score relative to those who identified as non-Hispanic White. Similarly, the analytic sample respondents who identified as Hispanic were more likely to rate their healthcare from their provides with a top box score relative to those who identified as non-Hispanic White. From a gender perspective (using the two gender identifications included in MEPS), the respondents in the analytic sample who identified as female were more likely to rate their healthcare from their providers with a top box score relative to those who identified as male.

In evaluating the model results under a more constrained top box score where only a score of 10 was included, the results aligned in some areas (the two significant Medicare-based findings remained significant) and differed in others (the uninsured finding (18–64) was no longer significant and in the constrained model, public based-insurance (18–64) and Medicare/other public (≥65) were both associated with higher odds of a top box score relative to private health insurance (18–64)). However, based upon the recommendation of AIR/RWJ [27] and as illustrated in an example included within an instruction manual for CAHPS [26], we continued with the 9–10 top box score method.

## 4. Discussion

How patients rate their healthcare experience has been an evaluation metric in healthcare payment models, including Medicare [8,9,10]. Accordingly, understanding how patients rate their healthcare is important from not only a patient experience perspective, but also from a reimbursement perspective. Research has illustrated that healthcare outcomes may differ by health insurance status [30,31]. This study found that how people rate their healthcare across all their doctors/providers may also be associated with their health insurance types. Specifically, it was estimated here that relative to respondents included in this study’s analytic model who were 18–64 years of age and had private health insurance, respondents that were 18–64 years of age who were without health insurance were less likely to rate the healthcare they received from all their providers as a top box score (a score of 9 or 10 on a 10-point scale where a 10 indicates the best possible care), while those with Medicare and those with Medicare/private health insurance who were 65 years of age and older were more likely to rate their healthcare in the top box score.

Hsiang et al. (2019) found in a meta-analysis that people with Medicaid encountered greater challenges than people with private health insurance in scheduling healthcare appointments [32]; a finding that could influence patient experience. However, in this study, there was no statistical difference among those who were 18–64 years of age with private health insurance relative to those who were 18–64 years of age with public health insurance. This finding aligns with Wray et al. (2021), who also did not find a statistical difference in satisfaction among people with private, employer-sponsored health insurance and those with Medicaid [33]. In some healthcare settings (e.g., emergency departments), patients may have similar patient experiences, which might explain why people 18–64 years of age with private health insurance and public health insurance may rate their healthcare similarly. Nevertheless, statistical differences were found in this study in terms of how people rated their healthcare between people that are 18–64 years of age with private health insurance and those that are 65 years of age or over with Medicare as well as those that are 65 years of age and older with Medicare/private health insurance coverage.

On a per member basis, people enrolled in Medicare (USD 13,490) and Medicaid (USD 8836) spent more money on health services in 2020 relative to the per member expenditure (USD 5862) among people who had employer-sponsored private health insurance [34]. Accordingly, if these expenditures correspond with utilization, people with Medicare may rate their healthcare from their providers higher due to a greater familiarity with the health services field. While an additional study is needed (one using a more comprehensive measure of utilization), the variables in the multiple logistic regression denoting respondents’ number of visits to a clinic or doctor’s office for care [24,25] illustrate that relative to just one visit to a doctor’s office or clinic for care, people with 2, 3, 4, or 5–9 visits tended to be less likely to rate their healthcare received as a top box score. Thus, it may be that fewer interactions with the healthcare system could result in higher ratings. The results on health status may support this as well. Relative to respondents included in the analytic sample who perceived their health status as poor, respondents who perceived their health status as excellent, very good, or good were all more likely to score their healthcare from their providers as a 9 or 10.

People who identify their health as less than excellent may have more complex health needs. The type of health insurance a person has can help determine how their complex healthcare needs are managed (e.g., the extent to which patients are required to obtain referrals and/or prior approvals before receiving care) which may then influence how they rate their healthcare from their providers. In addition, a limited provider network might influence how patients perceive their healthcare (e.g., they had to travel to see a specialist or a desired provider was “out-of-network”), which could help explain differences in how patients rate the healthcare from their providers. Considering the results for those without health insurance. Researchers have found a relationship between not having health insurance and lower health services utilization [35,36,37]. Although not examined here, there are a number of elements that may influence how people without health insurance perceive their healthcare once receiving healthcare services. For instance, people without health insurance could face greater restrictions and delays in care relative to others [38], which could influence how they perceive their care. In addition, the actual care people receive may differ by health insurance type [38,39,40], as illustrated by the findings of Asch et al. (2006) that there was a statistical difference between the recommended care received by people who do not have health insurance (53.7%) relative to people with Medicare (56.9%); conversely, there were not statistical differences in the amount of care received by people without health insurance and other groups in the Asch et al. study [40]. The stigma experiences of patients with public health insurance, as illustrated by Martinez-Hume et al. [41], may also explain how patients perceive the healthcare that they receive from their providers based upon health insurance type. We did not evaluate such elements and others in this study, which is a study limitation. Future research should evaluate the possible mechanisms that help explain why the health insurance type may be associated with how patients rate their healthcare from their providers. However, we did find support for the concept that health insurance type may be associated with how people rate their healthcare from their providers. Patient experience may influence both healthcare outcomes as well as the financial outcomes of a healthcare facility. Understanding factors that influence patient experience can thus assist stakeholders in creating performance improvement initiatives to address these factors and improve their patient experience metrics.

## 5. Conclusions

Relative to people who are 18–64 years of age with private health insurance, it was found here that people 18–64 years of age without health insurance were less likely to rate the healthcare they received from their doctors/providers with a top box score while people aged 65 years or over with Medicare or with Medicare/private health insurance were more likely to rank the healthcare they received from their doctors/providers as a top box score. Possible factors underlying these associations were not evaluated. Accordingly, as a next step, stakeholders should evaluate possible factors that may help explain the association, found here, between how patients rate their healthcare and their health insurance types (e.g., different care management programs).

Understanding the association between how patients rate their healthcare and health insurance type may help improve patient experience and close a possible gap between patient healthcare rating scores based on people’s health insurance types. Furthermore, some evidence illustrates that patient outcomes and experiences are not mutually exclusive but rather inter-related. Thus, improving patient experience (how they rate their healthcare) may also improve patient outcomes.

## Figures and Tables

**Table 1:** Weighted means/percentages ^1^ of the model variables by health insurance type.

	All	18–64 Years of Age			Years of Age ≥ 65				
		Private	Public	Un- Insured	Medicare	Medicare /Private	Medicare/ Other Public	Un- Insured	No Medicare/Other Public
Patient healthcare rating (top box score) ^2^	49.67%	46.37%	42.57%	38.36%	60.18%	62.36%	53.69%	87.58%	63.95%
Age ^3^ (mean)	51.84	43.26	43.25	41.87	74.55	73.18	74.68	72.84	67.85
Female	57.20%	57.37%	62.76%	49.34%	61.88%	51.77%	50.92%	42.13%	39.14%
Race									
Non-Hispanic African American/Black	10.06%	8.94%	19.31%	14.92%	7.04%	6.82%	13.26%	22.23%	15.67%
Non-Hispanic Asian	5.26%	6.15%	4.88%	1.68%	4.72%	2.76%	5.34%	13.22%	18.01%
Hispanic	11.65%	10.92%	18.67%	36.76%	7.51%	3.84%	22.14%	64.55%	5.22%
Non-Hispanic other race or multiple race	2.78%	2.71%	4.89%	2.52%	1.82%	1.82%	3.28%		2.22%
Non-Hispanic White	70.26%	71.27%	52.25%	44.12%	78.91%	84.76%	55.97%		58.88%
Marital status									
Married	55.36%	60.37%	30.23%	46.39%	52.80%	66.25%	33.84%	69.93%	48.06%
Widowed	7.68%	1.52%	3.84%	1.56%	26.70%	18.16%	27.08%	30.07%	9.54%
Divorced	12.20%	9.74%	17.86%	13.25%	15.18%	11.30%	24.93%		24.45%
Separated	1.80%	1.35%	4.83%	3.72%	1.01%	0.45%	4.52%		4.18%
Never married	22.95%	27.02%	43.23%	35.09%	4.31%	3.84%	9.63%		13.77%
Education									
Less than 12 years of education	10.46%	6.05%	24.00%	25.60%	12.59%	6.24%	32.57%	77.77%	4.27%
12 years of education	24.66%	20.34%	36.92%	30.55%	31.31%	24.56%	28.13%	22.23%	29.09%
At least some college	64.89%	73.61%	39.08%	43.84%	56.10%	69.20%	39.30%		66.64%
Income as % of the poverty level (mean)	488.30	563.58	182.70	302.03	417.29	599.78	235.12	174.57	670.73
Health status (self-reported)									
Poor	3.10%	1.33%	9.78%	2.47%	2.98%	2.98%	10.03%		
Fair	11.76%	7.54%	22.82%	10.73%	15.65%	12.78%	25.86%	40.91%	16.26%
Good	30.52%	28.04%	33.42%	37.22%	34.73%	32.26%	36.23%	36.67%	25.69%
Very good	35.39%	39.30%	22.85%	34.77%	32.82%	36.83%	19.60%	22.42%	21.31%
Excellent	19.24%	23.78%	11.13%	14.81%	13.82%	15.14%	8.28%		36.75%
Visits to a clinic or doctor’s office for care									
1 visit	25.91%	30.72%	22.93%	54.20%	14.66%	15.38%	11.88%	23.22%	21.94%
2 visits	22.07%	23.32%	21.61%	18.44%	20.63%	19.90%	19.64%	35.87%	15.26%
3 visits	15.09%	15.09%	15.18%	12.75%	15.21%	15.56%	13.49%	40.91%	24.19%
4 visits	11.65%	10.50%	11.03%	6.38%	15.95%	14.47%	12.89%		12.06%
5–9 visits	16.17%	13.65%	16.31%	5.95%	22.00%	21.87%	24.41%		17.91%
10 or more visits	9.10%	6.72%	12.94%	2.28%	11.54%	12.81%	17.69%		8.64%
Insurance ^2,4^									
Private (18–64 years of age)	56.94%	100.00%							
Public (18–64)	12.12%		100.00%						
Uninsured (18–64)	2.73%			100.00%					
Medicare (18–64)	10.29%				100.00%				
Medicare, private (≥65)	13.90%					100.00%			
Medicare and other public (≥65)	3.69%						100.00%		
Uninsured (≥65)	0.03%							100.00%	
No Medicare, other public (≥65)	0.29%								100.00%

Notes: ^1^ Rounded weighted means/percentages calculated from MEPS 2019 data [24,25] with complex survey design adjustments. In the subpopulation calculations, some strata were omitted if subpopulation members were not included in the strata. ^2^ Percentage of people included in this study’s sample from the 2019 MEPS data who rated the healthcare they received from all providers as a 9 or 10 on a 10-point scale where 10 indicates the best care. ^3^ Age is top coded at 85 years in MEPS. ^4^ The health insurance groups were provided in the MEPS data and are mutually exclusive groups.

**Table 2:** Multiple logistic regression: possible predictors of a “Top Box” score of how patients rate their healthcare from their providers ^1^.

	OR ^2^	SE	*p*	95% CI.L	95% CI.U
Age ^3^	1.01	0.00	<0.001	1.01	1.02
Female	1.10	0.04	0.016	1.02	1.19
Race (base group: White)					
Non-Hispanic African American/Black	1.25	0.09	0.003	1.08	1.45
Non-Hispanic Asian	0.85	0.09	0.128	0.68	1.05
Hispanic	1.51	0.11	<0.001	1.31	1.75
Non-Hispanic other race or multiple race	0.83	0.11	0.166	0.65	1.08
Marital status (base group: married)					
Widowed	1.01	0.09	0.898	0.85	1.21
Divorced	0.89	0.06	0.106	0.77	1.03
Separated	0.93	0.13	0.636	0.70	1.24
Never married	0.93	0.07	0.287	0.81	1.07
Education (base group: at least some college)					
Less than 12 years of education	1.05	0.08	0.560	0.90	1.22
12 years of education	1.03	0.06	0.556	0.93	1.15
Income as % of the poverty level	1.00	0.00	0.867	1.00	1.00
Health status (self-reported) (base group: poor)					
Fair	1.11	0.14	0.371	0.88	1.42
Good	1.48	0.18	0.001	1.17	1.88
Very good	2.34	0.29	<0.001	1.84	2.98
Excellent	3.98	0.51	<0.001	3.10	5.12
Visits to a clinic or doctor’s office for care (base group: 1 visit)					
2 visits	0.74	0.05	<0.001	0.64	0.85
3 visits	0.70	0.05	<0.001	0.60	0.81
4 visits	0.80	0.06	0.007	0.69	0.94
5–9 visits	0.82	0.06	0.009	0.70	0.95
10 or more visits	0.95	0.08	0.514	0.80	1.12
Insurance (base group: private (18–64)) ^3,4^					
Public (18–64)	1.07	0.08	0.348	0.93	1.25
Uninsured (18–64)	0.69	0.10	0.015	0.51	0.93
Medicare (≥65)	1.34	0.12	0.002	1.12	1.61
Medicare, private (≥65)	1.48	0.12	<0.001	1.25	1.74
Medicare and other public (≥65)	1.14	0.14	0.309	0.89	1.45
Uninsured (≥65)	6.72	6.87	0.064	0.89	50.54
No Medicare, other public (≥65)	1.56	0.53	0.192	0.80	3.07

Notes: ^1^ People, included in this study’s sample from the 2019 MEPS data [24,25], who rated the healthcare they received from all providers as a 9 or 10 on a 10-point scale where 10 indicates the best care. 95% CI.L = 95% confidence interval, lower limit. 95% CI.U = 95% confidence interval, upper limit. ^2^ Rounded data, odds ratios computed from a logistic regression using survey commands within Stata 17 [29] to adjust for the complex survey design of the MEPS dataset. ^3^ Age is top-coded at 85 years in the MEPS data. ^4^ The health insurance groups were provided in the MEPS data and are mutually exclusive groups.

## Data Availability

This study used publicly available data.
